# Direct Conjugation of NEDD8 to the N-Terminus of a Model Protein Can Induce Degradation

**DOI:** 10.3390/cells10040854

**Published:** 2021-04-09

**Authors:** Kartikeya Vijayasimha, Marilyn Vo Tran, Amy L. Leestemaker-Palmer, Brian P. Dolan

**Affiliations:** Carlson College of Veterinary Medicine, Oregon State University, Corvallis, OR 97331, USA; vijayask@oregonstate.edu (K.V.); tranmar@oregonstate.edu (M.V.T.); Amy.Palmer@oregonstate.edu (A.L.L.-P.)

**Keywords:** ubiquitination, NEDD8, protein degradation, proteasome

## Abstract

While the role of ubiquitin in protein degradation is well established, the role of other ubiquitin-like proteins (UBLs) in protein degradation is less clear. Neural precursor cell expressed developmentally down-regulated protein 8 (NEDD8) is the UBL with the highest level of amino acids identified when compared to ubiquitin. Here we tested if the N-terminal addition of NEDD8 to a protein of interest could lead to degradation. Mutation of critical glycine residues required for normal NEDD8 processing resulted in a non-cleavable fusion protein that was rapidly degraded within the cells by both the proteasome and autophagy. Both degradation pathways were dependent on a functional ubiquitin-conjugation system as treatment with MLN7243 increased levels of non-cleavable NEDD8-GFP. The degradation of non-cleavable, N-terminal NEDD8-GFP was not due to a failure of GFP folding as different NEDD8-GFP constructs with differing abilities to fold and fluoresce were similarly degraded. Though the fusion of NEDD8 to a protein resulted in degradation, treatment of cells with MLN4924, an inhibitor of the E1 activating enzyme for NEDD8, failed to prevent degradation of other destabilized substrates. Taken together these data suggest that under certain conditions, such as the model system described here, the covalent linkage of NEDD8 to a protein substrate may result in the target proteins degradation.

## 1. Introduction

Intracellular protein turnover is an important physiological process. By degrading a subset of proteins and synthesizing different ones, a cell can alter its function in response to environmental changes [1] and remove old or damaged proteins, which prevents their accumulation within cells and can lead to toxicity [2,3,4,5]. Protein degradation is also necessary for the recycling of amino acids [6,7,8] and for alerting the adaptive immune system to the presence of intracellular infections via the direct major histocompatibility (MHC) class I antigen presentation pathway [9,10,11]. A prominent pathway of protein degradation involves the proteasome, a multi-subunit protein complex containing trypsin-like, chymotrypsin-like, and caspase-like protease activity [12]. To prevent non-specific protease activity, proteins intended for proteasome-mediated degradation are modified by the addition of a small protein termed ubiquitin [13,14,15]. Once ubiquitinated, a targeted protein can be degraded via the proteasome or additional ubiquitin proteins can be added to the target protein leading to its subsequent destruction by the proteasome [16,17,18].

There are several other proteins within eukaryotic cells that are similar to ubiquitin and are termed ubiquitin-like proteins (UBLs) [19,20] and, like-ubiquitin, can be post-translationally added to a variety of targets proteins resulting in changes to the proteins’ functions [21,22]. Unlike ubiquitin, most UBL-protein modifications do not result in a protein’s destruction but rather alter protein function or localization [23]. There are a few exceptions to this “rule” though, which suggest that in certain circumstances, UBL modification may target a protein for destruction. FAT10 is a UBL with two ubiquitin domains and fusion of FAT10 to otherwise stable proteins resulted in their rapid degradation in the proteasome [24]. In addition, FAT10 can bind to regulatory units on the proteasome allowing for FAT10 conjugates to be degraded [25].

The UBL with the greatest similarity to ubiquitin is neural precursor cell expressed developmentally down-regulated protein 8 (NEDD8). Similar to ubiquitin, the covalent linkage of NEDD8 to a target substrate requires an E1-activating enzyme, an E2-conjugating enzyme, and an E3 ligase. NAE1 is the E1 activating enzyme for NEDD8 [26,27], while both UBE2M and UBE2F are NEDD8 E2-conjugating enzymes [28,29,30]. Multiple E3 ligases have been identified, which can facilitate the transfer of NEDD8 to its substrate (reviewed in [31,32]). NEDDylation can be reversed through the action of the COP9 signalosome [33], which can remove NEDD8 from substrates.

The canonical function of NEDD8 is to modify the scaffolding cullin family of proteins. Cullins associate with RING-box proteins to form Cullin-Ring-Ligases (CRLs), the largest family of E3 ubiquitin ligases. The modification of cullins by a single NEDD8 domain brings about a conformational change that facilitates the transfer of ubiquitin from a recruited E2-ubiquitin conjugating enzyme to the substrate of the CRL [34,35,36]. However, NEDD8 is known to covalently link to other proteins, such as TRAF6, VHL, p53, BCA3, and ribosomal protein L11 to name a few [37,38,39], and the consequences of protein NEDDylation are often unknown [31].

Several recent reports have highlighted the potential for non-canonical NEDDylation to result in a target protein’s destabilization and destruction. The first substrate identified that could be destabilized and destroyed following NEDD8 conjugation was the epithelial growth factor receptor (EGFR) [40,41]. Other proteins soon joined the list of substrates that could be destabilized and degraded following NEDDylation including HDAC, JunB, SMF2, c-Src, SRSF3, Ajuba, and E2F-1 [42,43,44,45,46,47,48,49]. In some instances, degradation was mediated by the ubiquitin-proteasome system [42,43,44,47,48,49]. Additionally, the proteasome-associated protein, NEDD8 ultimate buster 1 (NUB1), has been shown to interact with NEDD8-modified proteins, facilitating their degradation [50]. Considering its similarity to ubiquitin, it is possible that non-canonical NEDD8-modification can serve as a signal for protein degradation.

Here we test this hypothesis by creating a non-cleavable form of NEDD8 appended to the N-terminus of GFP. Fusion of ubiquitin and UBLs to the N-terminus of a protein of interest is a powerful tool for identifying UBLs, such as FAT10, that can target a protein for degradation [24,51]. Ubiquitin-fusion has also been extensively used for the study of direct antigen presentation of peptides via the MHC class I antigen presentation pathway [52,53,54,55]. We find the fusion of NEDD8 resulted in the rapid degradation of GFP by both autophagy and the proteasome. Degradation required ubiquitination and was partially dependent on the action of NUB1. Interestingly, the N-terminal addition of NEDD8 to GFP prevented fluorescence in eukaryotic cells, though restoring protein folding and fluorescence was not sufficient to prevent rapid protein degradation. While the addition of NEDD8 to a protein could serve as a signal for protein degradation, eliminating the NEDD8 activity in the cell by treatment with MLN4924 did not alter the overall degradation of another rapidly degraded protein. These data indicate that under certain circumstances, the non-canonical addition of NEDD8 to a target protein may result in the proteins’ destruction.

## 2. Materials and Methods

### 2.1. Plasmids, Antibodies, and Reagents

All primers were from Integrated DNA Technologies (IDT, Coralville, IA, USA) and PCR reactions amplified using a Veriti thermocycler (Applied Biosystems, Foster City, CA, USA). To generate pCAGGS-IRES-Thy1.1, we PCR amplified the IRES-Thy1.1 cassette from pMSCV Thy1.1 using the forward primer 5′-ATATATGCTAGCTGATCAGCGGCCGCACCGGTTTCGAACCCCCCCCCCTAACGTTACTGGCCGAA-3′ and reverse primer 5′-ATAAGATCTTCACAGAGAAATGAAGTCCAGGGCTTGGAGGAG-3′. The resulting PCR product was subcloned into pCAGGS using the NheI and BglII restriction sites and confirmed by Sanger DNA sequencing at the Oregon State University Center for Genome Research and Biocomputing. DNA sequences encoding fusion proteins (wildtype human NEDD8 or non-cleavable (NC) NEDD8) fused in frame with either enhanced GFP (hereafter GFP) or a cytosolic form of ovalbumin (hereafter OVA) were generated as Gene Blocks by IDT and contained an N-terminal EcoRI site and a C-terminal NheI site for subsequent cloning into pCAGGS-IRES-Thy1.1. For NC NEDD8-constructs, the three terminal glycines of NEDD8 were changed to alanine and fused in frame with the protein of interest. To generate the NC NEDD8-SL8-GFP construct, the amino acid sequence SIINFEKL was introduced between the terminal amino acid for NC NEDD8 and the first amino acid of GFP. Sanger sequencing was used to confirm proper cloning. For expression in E.coli, NEDD8-GFP fusion constructs were PCR amplified with the forward primer 5′-GCTGTACCATGGGTAGGAGGACAGCTATGCTAATTAAAGTGAAGA-3′ and the reverse primer 5′-GTCTACTACTTGTACAGCTCGTCCAGAATTCTACGAA-3′ to introduce a Shine-Dalgarno sequence upstream of the ATG start codon and NcoI and EcoRI restriction sites. PCR amplicons were cloned into pET-6X HIS-C terminal prokaryotic expression vector. For prokaryotic expression of GFP alone, the same procedure was followed except the following forward primer 5′-GCTGTACCATGGGTAGGAGGACAGCTATGGCCCGGGATCCACCGGTCGC-3′ was used in conjunction with the previously described reverse primer. Plasmids for transfection were prepared using the Qiagen HiSpeed Midi Plasmid Purification kit according to the manufacturer’s instructions. The following mouse monoclonal antibodies were utilized: anti-GFP (clones 7.1 and 13.1, Roche), anti-Thy1.1 (clone HIS51, eBiosciences, San Diego, CA, USA), anti-p97 (clone 58.13.3, Fitzgerald, Acton, MA, USA), and anti poly-ubiquitin (clone FK2, Enzo Life Sciences, Farmingdale, NY, USA). The following rabbit polyclonal antibodies were also used: anti beta-actin (Bethyl Laboratories, Montgomery TX, USA, A300-485A, Lot#3), anti-NUB1 (Cell Signaling, Danvers MA, USA, 14810S, Lot#1), anti-ATG7 (Cell Signaling, D12B11, Lot#3), and anti-NEDD8 (Cell Signaling 2745S, Lot#3). Infrared dye-coupled secondary antibodies (goat anti-mouse or goat anti-rabbit) were from LI-COR (Lincoln, NE, USA). The Ubiquitin E1 inhibitor MLN7243 (ChemieTek, Indianapolis, IN, USA) and NEDD8 E1 inhibitor MLN4924 (Millipore, Burlington, MA, USA) were dissolved in DMSO and used at a final concentration of 10 μM. MG132 (Millipore) and Epoxomicin (Enzo) were dissolved in DMSO and used at 10 μm for proteasomal inhibition of cells. The autophagy inhibitor 3-methyladenine (3MA) was from Millipore and used at a final concentration of 50 μM while Bafilomycin was from Tocris (Bristol, UK) and used at a final concentration of 0.1 µM. Cycloheximide (CHX, Millipore) was dissolved in DMSO and used at 25 µg/mL to block protein translation. Ammonium chloride (used at a final concentration of 5 mM) was from Sigma (St. Lois, MO, USA). Recombinant GFP was from Sino Biological (Wayne, PA, USA). Shield-1 was obtained from Clontech and was used at a final concentration of 2.5 μM.

### 2.2. Cell Culture and Transfection

EL4, EL4/SCRAP-GFP, and MCF7 cell lines have been previously described [56,57] and were a kind gift from Dr. Jonathan Yewdell (NIH). All cells were grown in RPMI 1640 supplemented with 10 mM HEPES, 20 mM Glutamax, and 7.5% fetal calf serum (all from Thermo Fisher, Waltham, MA, USA) and were incubated at 37 °C and 6% CO_2_. Manual cell counting with a hemocytometer was used to determine the concentration and number of cells in subsequent experiments. The creation of EL4 and MCF7 cell lines stably expressing NEDD8-GFP and NC NEDD8-GFP recombinant proteins was accomplished by the transfection of the pCAGGS-IRES-Thy1.1 expressing either protein, followed by magnetic sorting. Briefly, 1 × 10^6^ cells were transfected with 8 μg ScaI-linearized plasmid using the Amaxa Nucleofector (Lonza, Basel, Switzerland) SF kit on program DS-113 (for EL4 cells) or FF-120 (for MCF7 cells). Transfected cells were allowed to recover for 2 days and were then labeled at 4 °C with anti-Thy1.1 antibody (clone HIS51, eBiosciences) for 30 min in PBS buffer containing 0.1% bovine serum albumin (BSA, Amresco, Solon, OH). Cells were then washed in buffer and incubated with anti-mouse IgG microbeads beads (Miltenyi Biotech, Bergisch Gladbach, Germany) for 30 min at 4 °C, washed, and resuspended in 1 mL buffer. Thy1.1 positive cells were selected by passing cells over LD columns using the MidiMACS separator system per the manufacturer’s instructions. Eluted cells were added to warm media containing antibiotics. Sorted cells were cultured for 48–72 h before being sorted again. This process is repeated until a population with >90% Thy1.1 expression was obtained.

### 2.3. Flow Cytometry

Approximately 1 × 10^6^ cells/mL were harvested and washed with Hank’s balanced salt solution (HBSS) supplemented with 0.1% BSA. Cells were then resuspended with 0.1% BSA/HBSS containing PE-Cy5.5 coupled anti-Thy1.1 antibodies (clone HIS51, eBiosciences), or isotype control and incubated for 30 min at 4 °C. The cells were washed again by resuspending with 0.1% BSA/HBSS before being analyzed with an Accuri C6 benchtop flow cytometer (BD Biosciences, San Jose, CA, USA) as previously described [58]. Appropriate filter sets were used to detect GFP fluorescence or PE-Cy5.5 fluorescence.

### 2.4. siRNA Knockdown

One million EL4/NC NEDD8-GFP cells were harvested and transfected with 1 μM mouse Nub1 or Atg7 siRNA (Dharmacon, Lafayette, CO, USA), or Scrambled siRNA (as a negative control) using the Amaxa Nucleofector as described above. Transfected cells were incubated in growth media for 48 h before being processed for Western blot analysis.

### 2.5. Western Blotting

Cells were harvested and resuspended at 1 × 10^6^ cells per 100 μL of cold PBS supplemented with 0.5% Triton X-100 containing protease inhibitor (Complete protease inhibitor cocktail, Roche, Basel Switzerland) and were incubated on ice for 30 min. The solution was centrifuged at 11,000× *g* (at 4 °C) for 5 min. Bolt LDS buffer (Thermo Fisher) was added to the supernatant at one-fourth of the volume of the cell lysate along with 10 μM DTT. The solution was heated at 95 °C for 10 min before being resolved by SDS-PAGE using a 4–12% Bolt Bis-Tris gel (Thermo Fisher) and transferred to a nitrocellulose membrane (Thermo Fisher). Membranes were then blocked with a 5% solution of dehydrated milk in TBST (Tris-buffered saline, 0.1% Tween 20) for 40 min. Primary antibodies (1:1000–1:5000) and secondary antibodies (1:10,000) were diluted in 0.5% milk in TBST. Membranes were incubated with primary antibodies overnight, washed with TBST, and incubated with secondary antibodies for 1 h, followed by two 5-min washes in TBST and one rinse in deionized water. Membranes were analyzed using an infrared imager (LI-COR Odyssey) and blots quantified by densitometry using instrument software (Image Studio Lite, LI-COR Biosciences). Relevant bands were determined based upon either the predicted size of the protein or by its presence/absence in control experiments. All Western blot images were cropped to show relevant bands. Adjustments to the brightness or the contrast were applied uniformly to the entire image. Such alterations did not change the intensity values obtained by the instrument software.

*TUBE-isolation Assay.* The Tandem Ubiquitin Binding Entities kit (TUBE, Life Sensors, Malvern, PA, USA) was used to analyze polyubiquitinated products in transfected cells. The assay was conducted as per the manufacturer’s instructions. Briefly, 1 × 10^7^ cells were harvested and washed by centrifugation and resuspension in PBS. The washed cell pellet was then resuspended in cold lysis buffer, consisting of 100 µL Triton X 100, one protease inhibitor tablet (Roche), 10 μM MG132, and 200 µL N-ethylmaleimide (NEM, Thermo Fisher) made up to 10 mL with PBS, for 30 min on ice. The lysate was then centrifuged at 10,000× *g* for 10 min at 4 °C and a portion of the supernatant was saved as the pre-clear analysis. TUBEs were prepared by washing twice in cold lysis buffer and resuspended in cold lysis buffer equivalent to the starting volume of TUBEs. To isolate poly-ubiquitin proteins, 75 μL of prepared TUBEs were added to 500 μL of cell lysate. The TUBE-lysate mixture was incubated at 4 °C for 1 h while being constantly mixed. The samples were centrifuged at 5000× *g* and an aliquot of supernatant was saved for post-clear analysis. Beads were washed three times with 700 μL cold lysis buffer. Finally, the TUBEs were resuspended in 40 μL lysis buffer, 20 μL BOLT sample buffer, and 2 μL of 1 M DTT, and heated at 95 °C for 10 min. The solution was centrifuged and the supernatant was analyzed by Western blot analysis.

### 2.6. Prokaryotic Protein Expression

For analysis of NEDD8-GFP fusion products in a prokaryotic expression system, pET-6X His-C-terminal plasmids containing either NC NEDD8-GFP, NEDD8-GFP, or GFP alone, were cloned into INVITROGEN One Shot™ BL21 competent cells and plated onto LB plates containing ampicillin overnight. Individual colonies were selected and propagated overnight in LB broth containing ampicillin and used to seed a 5 mL culture the following day. The new culture was grown to log growth phase prior to GFP analysis. One-hundred microliters of the bacterial culture was analyzed by flow cytometry for GFP expression, while 75 μL of the culture was mixed with 25 μL of Bolt LDS sample buffer and boiled for 20 min in the presence of 1 μM DTT. Bacterial lysate was then resolved by SDS-PAGE and Western blot analysis for GFP as described above.

### 2.7. Statistical Analysis

All statistical analysis was carried out using GraphPad Prism software (San Diego, CA, USA). Protein half-lives were calculated using one-phase decay. Statistical significance between treatments was determined using the Student t-test. Differences were considered significant at *p* < 0.05.

## 3. Results

### 3.1. Non-Cleavable NEDD8-GFP Fails to Accumulate in EL4 Cells

**Figure 1:**
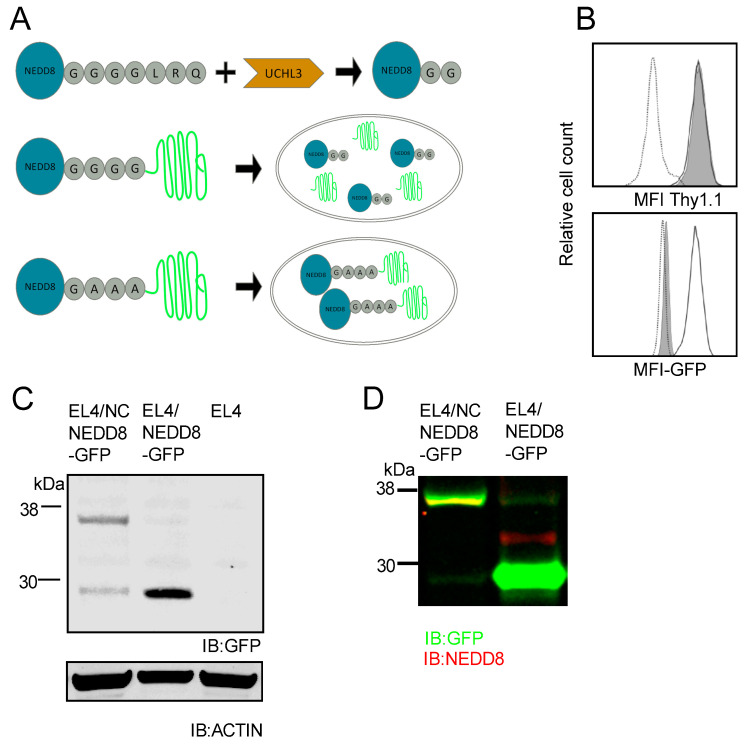
Fusion of NEDD8 to GFP leads to decreased GFP in stably transfected cells. (**A**) Cartoon representation of the components of the constructs. During normal NEDD8 processing, UCH-L3 cleaves between the second and third glycine residues releasing the active NEDD8 molecule. NEDD8 fused to GFP should be cleaved in the same manner as the wildtype NEDD8 protein, however, mutation of the 3 glycine residues to alanine residues will result in a fusion protein where NEDD8 is bound, in a non-cleavable manner to GFP. (**B**) Each NEDD8-GFP construct was cloned into an IRES expression vector containing a Thy1.1 reporter DNA sequence. EL4 cells (dotted trace) were stably transfected with either wildtype and cleavable NEDD8-GFP (solid trace) or non-cleavable (NC) NEDD8-GFP constructs (shaded histograms). Each cell type was analyzed by flow cytometry for both Thy1.1 expression (top histogram) and GFP (bottom histogram). (**C**) Western blot analysis of EL4/NEDD8-GFP and EL4/NC NEDD8-GFP cell lysates. Following SDS-PAGE and blotting onto nitrocellulose, membranes were probed with either mouse monoclonal antibodies against GFP or rabbit poly-clonal antibodies against actin and appropriate secondary antibodies. (**D**) Two-color immunoblot of EL4/NEDD8-GFP and EL4/NC NEDD8-GFP cell lysates. After SDS-PAGE and blotting, nitrocellulose membranes were probed simultaneously with mouse monoclonal antibodies against GFP and rabbit polyclonal antibodies against NEDD8. Antigen-antibody complexes were detected using goat anti-rabbit IRDye 680 CW (red), and goat anti-mouse 800 CW (green) secondary antibodies. Yellow regions correspond to simultaneous fluorescence in both channels. All Western blot images were cropped to show relevant bands.

To understand the effects of NEDD8 binding to the N-terminus of non-canonical substrates, we utilized the existing NEDD8 processing machinery and created two NEDD8-GFP fusion products as depicted in Figure 1A. The four-glycine motif is recognized by UCHL3, which cleaves between the second and third glycine [59,60], activating NEDD8. Mutation of these residues to alanine will likely prevent proper processing leaving NEDD8 fused to GFP. The first construct is NEDD8-GFP, in which wildtype human NEDD8 was fused in frame with GFP. The second construct is termed non-cleavable NEDD8-GFP (NC NEDD8-GFP), where glycine amino acid residues in the sequence of NEDD8 are mutated to alanine (G76, G77, G78 to A76, A77, A78) and then fused with GFP. Both constructs were cloned into a eukaryotic expression vector containing an IRES promoter followed by DNA sequence encoding the cell surface marker Thy1.1. EL4 cells were stably transfected with these constructs and sorted using magnetic beads for Thy1.1 expression. Following two rounds of cell sorting, nearly equivalent Thy1.1 expression was detected in both cell lines, which were greater than 90% positive for Thy1.1 expression as determined using flow cytometry (Figure 1B). GFP fluorescence measurements via flow cytometry showed an increase in fluorescence in cells transfected with the wildtype NEDD8-GFP construct (Figure 1B, solid trace), as compared to parental cells (dotted trace) as expected. However, very low levels of GFP fluorescence were detected in cells transfected with NC NEDD8-GFP construct (Figure 1B, shaded histogram). Western blot analysis of cell lysates with antibodies specific for GFP revealed a faint band at 36 kDa in EL4/NC NEDD8-GFP cells and an even fainter band at 28 kDa, corresponding to the size of processed GFP (Figure 1C). A GFP-reactive band was detected at 28 kDa in EL4/NEDD8-GFP cells and the band was much more intense than the GFP band in the non-cleavable form of the construct (Figure 1C). Simultaneous blotting for both GFP and NEDD8 reveal that the 36 kDa band observed in the EL4/NC NEDD8-GFP lysate co-stained for both NEDD8 and GFP, indicating that the NEDD8 remains mostly fused to GFP, whereas the 28 kDa protein only stains for GFP (Figure 1D). These data demonstrate that the triple glycine to alanine mutation prevents processing of NEDD8 from the fused GFP protein and the non-cleaved form of the protein accumulates to a much lower extent in cells.

### 3.2. Non-Cleavable NEDD8-GFP Has a Metabolic Half-Life of Approximately 26 Minutes in EL4 Cells

**Figure 2:**
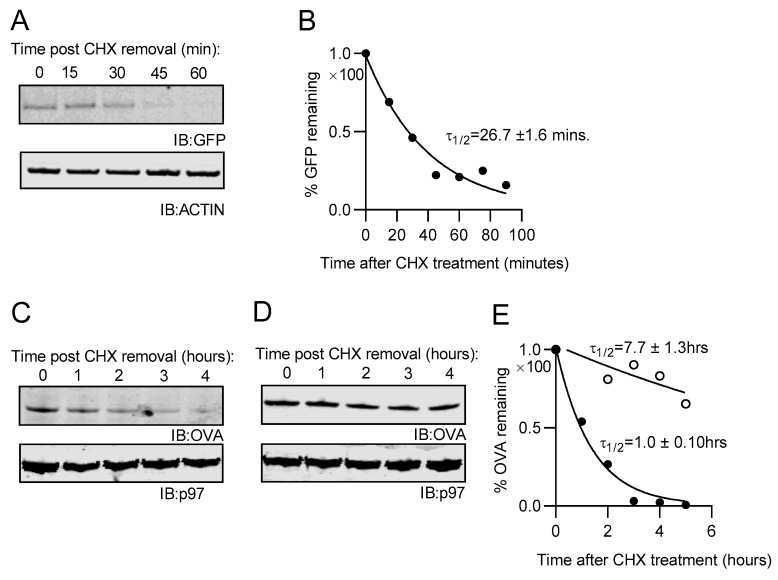
Half-life determination of the non-cleavable forms of NEDD8-GFP and NEDD8-OVA in EL4 cells. EL4 cells stably expressing model proteins were treated with CHX to prevent translation of new proteins. (**A**) Cell lysates of NC NEDD8-GFP were created at indicated times after CHX treatment and analyzed by Western blot analysis. (**B**) Single phase exponential decay analysis was used to calculate the half-life of NC NEDD8-GFP, after quantification of bands from Western blot. The mean half-life of GFP was calculated from three independent experiments to be 26.7± 1.6 min. (**C**,**D**) Cell lysates for EL4/NC NEDD8-OVA and EL4 cells stably expressing OVA alone were made at indicated times (after CHX treatment) and analyzed by Western blot. (**E**) Half-lives for NC NEDD8-OVA (black circles) and OVA-alone (white circles) were calculated as described before. The calculated half-life was 1 ± 0.1 h for NC NEDD8-OVA and 7.7 ± 1.3 h for OVA-alone. Data shown is from one of three independent experiments performed. Half-lives shown are mean values obtained from three independent experiments. All western blot images were cropped to show relevant bands.

Since the non-cleavable form of NEDD8-GFP was in low abundance within stably transfected cells, we suspected it may be undergoing rapid degradation. To test this, cells were incubated with CHX to prevent ongoing protein synthesis and analyzed by Western blot for GFP. Levels of NC-NEDD8-GFP within the cell decreased over time (Figure 2A). The half-life of NC NEDD8-GFP was determined by quantification of GFP-band intensity as determined by Western blot (Figure 2B). The calculated half-life for each of three independent experiments was averaged and the resulting half-life determined to be 26.7 ± 1.6 min. Considering the half-life of GFP is approximately 26 h [61], these data indicate that the non-cleavable form NEDD8 fused to GFP results in rapid degradation of the protein and explains why the protein is in low abundance compared to cleavable fusion protein. To determine if this was true for substrates other than GFP, we generated a non-cleavable NEDD8 construct fused to a cytosolic form of chicken ovalbumin (NC NEDD8-ova), which contains the same triple glycine to alanine mutation as described for our GFP constructs. Western blot analysis of stably transfected EL4 cells for ovalbumin revealed a rapid decrease in NC NEDD8-ova protein following CHX treatment (Figure 2C) when compared to cells expressing cytosolic ova alone (Figure 2D). The average half-life of NC NEDD8-ova from three independent experiments was calculated to be 1.1 ± 0.1 h, whereas the half-life of ovalbumin alone is 7.7 ± 1.3 h. These data indicate that the addition of NEDD8 to the N-terminus of a protein renders the protein subject to rapid degradation.

### 3.3. Non-Cleavable NEDD8-GFP Undergoes Proteasomal and Autophagosomal Degradation

**Figure 3:**
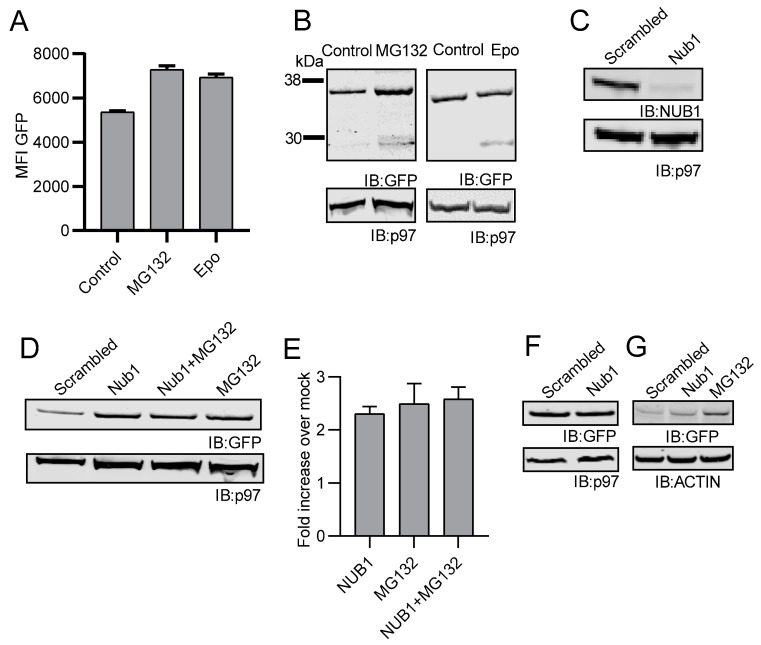
Non-cleavable NEDD8-GFP undergoes proteasomal degradation in a NUB1-dependent process. EL4 cells stably expressing NC NEDD8-GFP were treated with the proteasomal inhibitors MG132 or epoxomicin (epo) for 3 h and checked for NEDD8-GFP accumulation. (**A**) Flow cytometric analysis for GFP fluorescence of cells after proteasomal inhibition are compared with mock-treated cells as a control. The MFI of GFP for the total cell population is reported on the *y*-axis. (**B**) Cell lysates of EL4/NC NEDD8-GFP cells treated with MG132 or epoxomicin were made and analyzed by Western blot using mouse monoclonal antibodies against GFP and p97 with appropriate secondary antibodies. (**C**) Western blot analysis of EL4/NC NEDD8-GFP cells transfected with either scrambled siRNA oligomers or siRNA targeting NUB1. Cell lysates were probed for either NUB1 (top panel) or p97 as a loading control (bottom panel). (**D**) EL4/NC NEDD8-GFP cells were transfected with scrambled or NUB1-targeting siRNAs and cultured with or without MG132. Cell lysates were examined by Western blot analysis for GFP. (**E**) GFP signal obtained by Western blot was quantified in three independent experiments and calculated as fold-increase over mock-treated cells. Error bars represent the standard error. (**F**) GFP levels were analyzed by Western blot analysis in EL4/NEDD8-GFP cells upon NUB1 loss. (**G**) EL4/SCRAP cells were transfected with NUB1-specific or scrambled siRNAs are analyzed by Western blot for GFP. As a control, EL4/SCRAP cells transfected with scrambled siRNAs were treated with MG132 prior to cell lysis. All Western blot images were cropped to show relevant bands.

We next sought to determine how NC-NEDD8-GFP was degraded by cells. We first tested if proteasome inhibition would block NC NEDD8-GFP degradation. Inhibition of the proteasome for 3 h using either MG132 or epoxomicin resulted in an increased GFP signal as determined by both flow cytometry (Figure 3A) and Western blot (Figure 3B). Previous studies have reported that NEDD8 ultimate buster 1 (NUB1) is responsible for binding to NEDD8-protein conjugates as well as other UBL conjugates and may be responsible for transporting the conjugated substrates to the proteasome [50,51]. To test if NUB1 was involved in the degradation of non-cleavable NEDD8-GFP, we transfected EL4/NC NEDD8-GFP with siRNA oligomers targeting NUB1 or scrambled oligomers acting as a negative control. After siRNA transfection, cells were processed for Western blotting. NUB1 levels were greatly reduced following transfection with NUB1-specific siRNA oligonucleotides (Figure 3C), as determined by Western blot analysis. Following NUB1 depletion, the levels of NC NEDD8-GFP were increased in cells, and treatment of NUB1-depleted cells with MG132 failed to further increase levels of NC NEDD8-GFP (Figure 3D). Quantification of Western blots from three independent experiments demonstrates a 2–3-fold increase in GFP signal following either proteasome inhibition or NUB1 depletion (Figure 3E) and the increase in signal detected was statistically different from untreated cells (*p* < 0.05). These data indicate that NC NEDD8-GFP can be degraded by the proteasome in a NUB1-specific manner. No increase in GFP was seen when NUB1 was eliminated from EL4 cells expressing the wildtype NEDD8 fused to GFP, though levels of GFP were already quite high in these cells and unlikely to increase (Figure 3F). To determine if the action of NUB1 was specific for NEDD8-conjugated substrates, we depleted NUB1 from a second cell line expressing a rapidly degraded form of GFP. EL4/SCRAP cells [57] expressing a fusion protein consisting of GFP and a destabilization domain controlled by the action of a small molecule termed Shield-1 [62]. SCRAP-GFP is rapidly degraded in the absence of Shield-1. Binding of Shield-1 allows for proper folding of the fusion protein, leading to GFP fluorescence and an increase in total GFP, which can be measured by Western blot. This construct has been previously used to determine the role of protein stability in antigen presentation [57,63]. Loss of NUB1 in EL4/SCRAP cells did not result in increased levels of SCRAP-GFP in cells (Figure 3G), suggesting that NUB1 is not involved in the degradation of all rapidly degraded proteins, or that NUB1 depletion adversely affects proteasome function in general. As a control, EL4/SCRAP cells transfected with scrambled siRNAs were treated with MG132 for 2 h, resulting in a noticeable increase in SCRAP-GFP (Figure 3G). Taken together, these data suggest that NEDD8-conjugation can result in protein targeting to the proteasome via interaction with NUB1.

**Figure 4:**
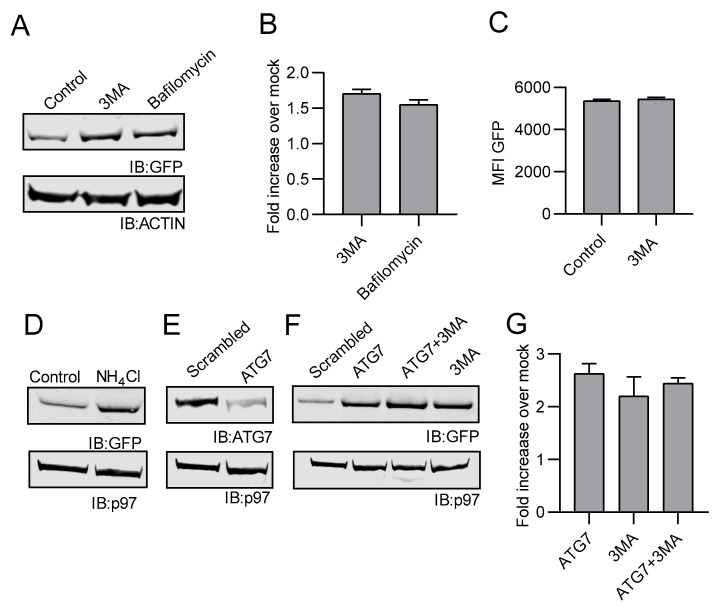
Non-cleavable NEDD8-GFP undergoes autophagosomal degradation. (**A**) Western blot analysis of EL4/NC NEDD8-GFP cells following a 5-h treatment with autophagy inhibitors 3-MA or bafilomcyin. Cell lysates of NC EL4/NEDD8-GFP cells and NC EL4/NEDD8-GFP treated with inhibitors were made and analyzed by Western blot using mouse monoclonal antibodies against GFP and actin with appropriate secondary antibodies. (**B**) The average fold increase in GFP following autophagy inhibition was determined by Western blot analysis is reported for three independent experiments. (**C**) Flow cytometric analysis for GFP fluorescence of cells after autophagosomal inhibition are compared with mock-treated cells. (**D**) Western blot analysis of EL4/NC NEDD8-GFP cells after overnight treatment with 5mM NH4Cl treatment. (**E**) Western blot analysis of EL4/NC NEDD8-GFP cells transfected with either scrambled siRNA oligomers or siRNA targeting ATG7. Cell lysates were probed for either ATG7 (top panel) or p97 as a loading control (bottom panel). (**F**) EL4/NC NEDD8-GFP cells were transfected with scrambled or ATG7-targeting siRNAs and cultured with or without 3-MA. Cell lysates were examined by Western blot analysis for GFP. (**G**) GFP signal obtained by Western blot was quantified in three independent experiments. The increase of NC/NEDD8-GFP for each treatment is shown as the fold-increase increases over mock-treated control. Error bars represent the standard error. All Western blot images were cropped to show relevant bands.

In addition to the proteasome, cells can also degrade protein via the autophagy pathway. Treatment of EL4/NC NEDD8-GFP cells with the autophagy inhibitors 3-MA or bafilomycin resulted in a measurable increase in GFP protein as determined by Western blot analysis (Figure 4A,B). Interestingly, no increase in GFP fluorescence was detected by flow cytometry when cells were treated with 3-MA (Figure 4C). The degradation of proteins via autophagy occurs when the autophagosome fuses with lysosomes allowing the lysosomal components to destroy the contents of the autophagosome. To determine if lysosomal components were necessary for autophagy-related degradation, we treated EL4/NC NEDD8-GFP cells with NH4Cl to prevent the acidification of lysosomes and analyzed cell lysates by Western blot analysis. Treatment with NH_4_Cl resulted in increased levels of GFP (Figure 4D). To further confirm the role of autophagy in the destruction of NC NEDD8-GFP, we transfected EL4/NC NEDD8-GFP cells with siRNAs targeting Atg7, an E1-like protein responsible for activating the ubiquitin-like molecules, Atg8 and Atg12, to facilitate autophagy [64,65]. Depleting Atg7 (Figure 4E) resulted in an increase in NC NEDD8-GFP (Figure 4F), providing genetic evidence that autophagy is one pathway that can degrade NEDD8-GFP. The addition of 3-MA to Atg7-depleted cells did not further increase levels of NC NEDD8-GFP, indicating that 3-MA treatment was specific for autophagy (Figure 4F). Similar to proteasome inhibition, inhibiting autophagy led to a 2–3-fold increase in NC NEDD8-GFP (Figure 4G) over mock-treated cells (*p* < 0.05). These data demonstrate that in addition to proteasome-mediated degradation, the non-cleavable form of NEDD8-GFP can also be degraded in the autophagosome.

### 3.4. MLN7243 Blocks Degradation of NC NEDD8-GFP

**Figure 5:**
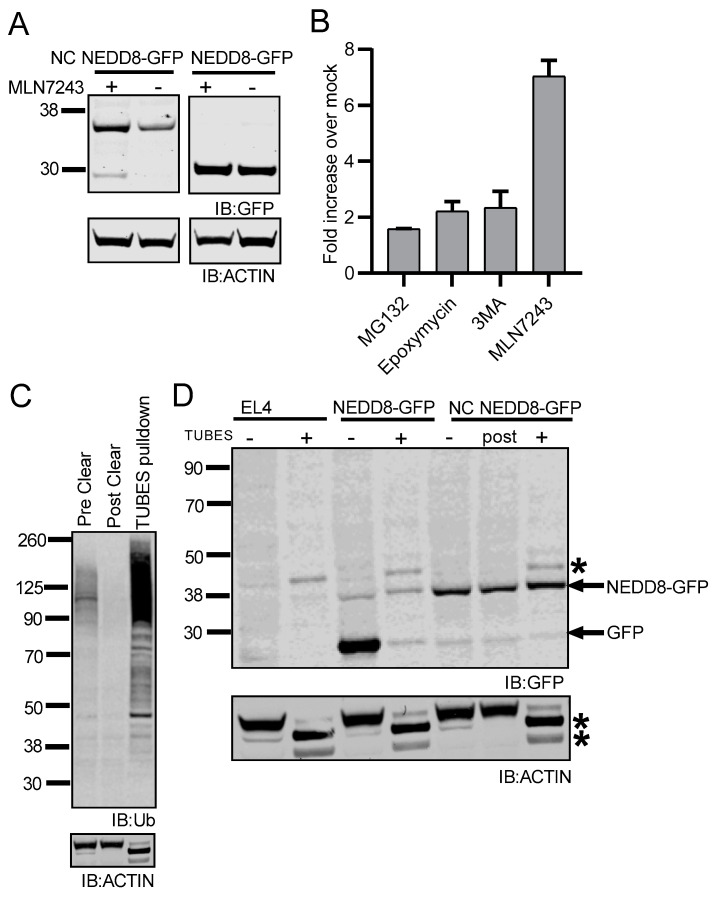
A functional ubiquitination system is required for NC NEDD8-GFP degradation. (**A**) EL4/NC NEDD8-GFP and EL4/NEDD8-GFP cells were treated with MLN7243 for 3 h to inactivate UBE1 preventing ubiquitination. Lysates were subjected to Western blot analysis for indicated proteins. (**B**) Western blot densitometry for GFP accumulation after treatment with MG132, Epoxomicin, 3MA, and MLN7243. GFP accumulation is shown as a fold increase over mock-treated cells. Values for MG132, Epoxomicin, and 3MA are from Figure 3 and Figure 4. (**C**) Western Blot analysis for poly-ubiquitinated proteins in EL4/NC NEDD8-GFP lysates prior to TUBES precipitation (labeled pre-clear), post-pull down (labeled post clear), and the TUBES precipitated proteins. Poly-ubiquitinated proteins were identified by staining with the ubiquitin-specific monoclonal antibody FK2. Actin (bottom panel) is shown as a control. (**D**) Poly-ubiquitinated proteins were isolated from EL4, EL4/NEDD8-GFP, and EL4/NC NEDD8-GFP cell lysates using TUBES and subjected to Western blot analysis with GFP monoclonal antibodies. Total cell lysates not subject to TUBES pull-down (labeled “-“) were compared to proteins eluted from TUBES (labeled “+”) and, in the case of NC NEDD8-GFP cell lysates, the remaining total cell lysate after removing poly-ubiquitinated proteins (labeled “post”). Bands corresponding to GFP and NC NEDD8-GFP are indicated. Bands marked by an asterisk (*) are contaminating bands reacting non-specifically to the presence of an antibody. All Western blot images were cropped to show relevant bands.

The addition of either a single or multiple ubiquitin molecules can act as a signal for proteasomal mediated-degradation [16,18,66]. To determine if degradation of the non-cleavable form of NEDD8-GFP required ubiquitination prior to degradation, we treated cells with MLN7243, a chemical inhibitor of the ubiquitin activating enzyme E1 (UBE1) [67,68]. EL4/NEDD8-GFP and EL4/NC NEDD8-GFP cells were cultured for 3 h with media supplemented with 10 μM MLN7243 and then processed for Western blotting. Probing cell lysates with anti-GFP antibodies showed a drastic accumulation of NC NEDD8-GFP in cell lysates after MLN7243 treatment (Figure 5A), compared to untreated cells. No, or minimal, accumulation of GFP was observed in EL4/NEDD8-GFP cell lysates after UBE1 inhibition (Figure 5A). Interestingly, the NC NEDD8-GFP band appeared more intense upon inhibition of ubiquitination compared to proteasome inhibition or inactivation of autophagy, as seen in Figure 3 and Figure 4. Densitometry quantification of GFP signal by Western blot following MLN7243 treatment demonstrated a statistically significant increase in MLN7243-treated cells compared to either proteasome or autophagy inhibition previously reported in Figure 3 and Figure 4. (Figure 5B, *p* < 0.05). To determine if NC-NEDD8-GFP was poly-ubiquitinated prior to degradation, we utilized tandem ubiquitin binding entities (TUBEs) beads to isolate poly-ubiquitinated proteins from EL4, EL4/NEDD8-GFP, and EL4/NC NEDD8-GFP cell lysates. TUBESs can recognize and bind to proteins modified with ubiquitin chains, including lysine 48 and lysine 63-linked ubiquitin chains [69]. Lysates were incubated with TUBEs beads, and proteins bound to the beads were eluted and subjected to Western blotting with monoclonal antibody FK2, which can recognize all mono- and poly-ubiquitinated substrates linked through any of the lysine residues. TUBEs beads were able to isolate nearly all detectable poly-ubiquitinated proteins from EL4/NC NEDD8-GFP cell lysates (Figure 5C), as almost no detectable poly-ubiquitinated proteins remained in the lysate after the pull-down. As a negative control, we show that actin levels remain unchanged in the lysate after TUBEs pull down and that actin itself is barely detectable in the TUBE-isolated fraction (Figure 5C). NC NEDD8-GFP was present in the TUBE-isolated proteins, indicating that TUBEs had a slight affinity to NC NEDD8-GFP (Figure 5D), though some NC NEDD8-GFP remained in the non-bead-bound fraction (labeled “post” in Figure 5D). We also detected the presence of very faint bands coinciding to NC NEDD8-GFP + 16 kDa (Figure 5D), which may correspond to NC NEDD8-GFP decorated with two ubiquitin moieties (8 kDa each). The presence of a contaminating band at 44 kDa (which is present in the TUBE pull-down in EL4 lysates, Figure 5D) made visualization of a NEDD8-GFP + 1 ubiquitin (8 kDa) band impossible. However, no higher molecular weight forms of NC NEDD8-GFP were detected, suggesting a lack of poly-ubiquitination of the NC NEDD8-GFP substrate. Taken together, these data suggest that the activity of a functional ubiquitination system is important for NC NEDD8-GFP degradation, but may not involve poly-ubiquitination of the substrate.

### 3.5. Fusion of NEDD8 to GFP Prevents GFP Fluorescence

**Figure 6:**
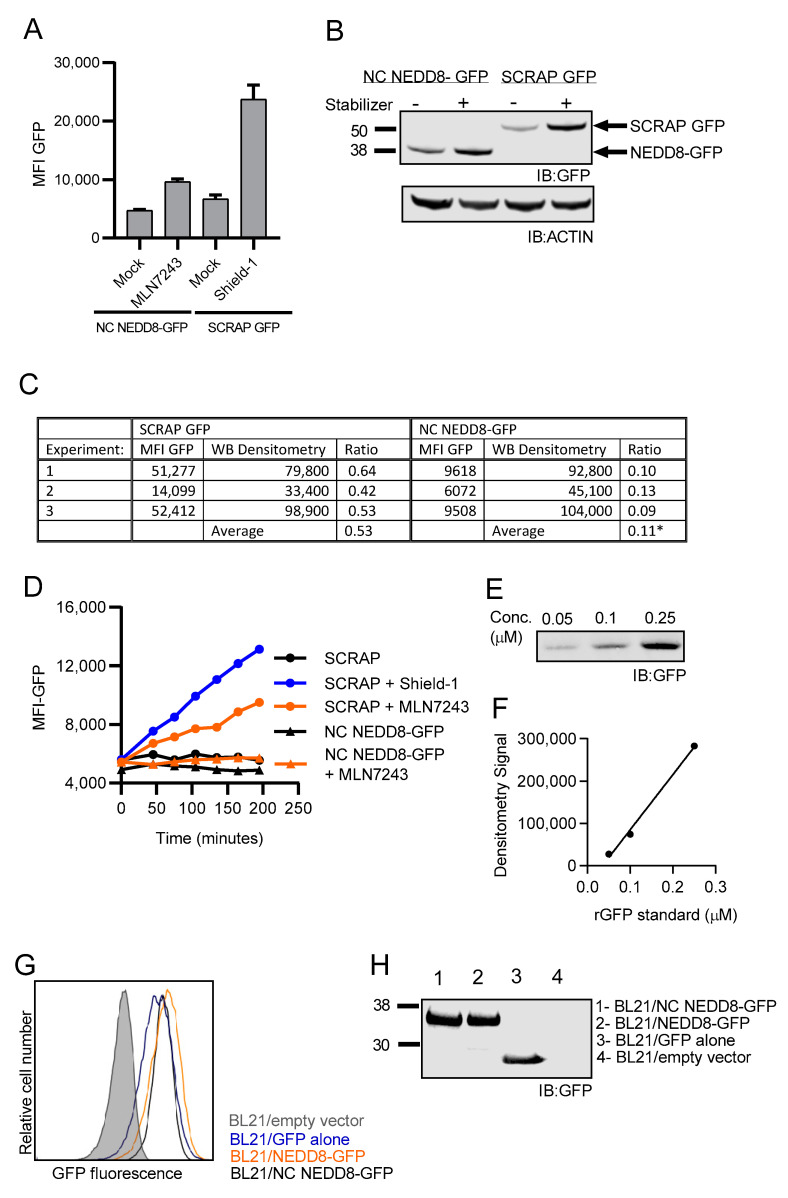
Fusion of NEDD8 to GFP prevents proper folding of GFP. (**A**) GFP fluorescence as determined by flow cytometry in EL4/NC NEDD8-GFP cells and EL4/SCRAP-GFP cells after treatment with chemical stabilizer (Shield-1 or MLN7243 respectively) for 3 h is shown. The average MFI of the cellular population from three independent measurements is reported on the *y*-axis. (**B**) Western blot analysis for GFP accumulation after treatment of EL4/NC NEDD8-GFP and EL4/SCRAP-GFP cells with their stabilizers, Shield-1 or MLN7243, respectively, for 3 h. (**C**) The ratio of fluorescent GFP signal obtained by flow cytometry (MFI GFP) to total GFP protein obtained by Western blot (WB Densitometry) for both SCRAP-GFP and NC NEDD8-GFP after treatment with stabilizer was calculated for three independent experiments and was statistically different (* *p* < 0.05). (**D**) Comparison of increased fluorescence of EL4/SCRAP-GFP cells and EL4/NC NEDD8-GFP cells after stabilizer treatment. Mean GFP fluorescence intensity of EL4/SCRAP-GFP cells (black circle), EL4/SCRAP-GFP cells treated with Shield-1 (blue circle) or MLN7243 (orange circle), and EL4/NC NEDD8-GFP (black triangle) and EL4/NC NEDD8-GFP treated with MLN7243 (orange triangle) were measured by flow cytometry at the indicated time points. (**E**) Representative Western blot of GFP standards with the concentration of recombinant GFP standard indicated. (**F**) Standard curve for the Western blot shown, (**E**) demonstrating the linearity of the signal determined by densitometry. (**G**) GFP fluorescence of *E. coli* expressing NC NEDD8-GFP (black trace), NEDD8-GFP (orange trace), GFP (blue trace), or empty cloning vector (shaded histogram) as determined by flow cytometry. (**H**) Western blot for GFP from the indicated cell lysates of *E. coli* from part (G) above. All Western blot images were cropped to show relevant bands.

It was surprising that despite a noticeable increase in GFP intensity by Western blot upon inhibition of protein degradation, very little GFP fluorescence was detected in cells, considering the fact that measuring GFP fluorescence by flow cytometry is far more sensitive than examining total GFP content in a cell lysate by Western blot. We therefore compared GFP fluorescence (measured by flow cytometry) and total GFP levels as determined by Western blot densitometry measurements between EL4/NC NEDD8-GFP and EL4/SCRAP-GFP cells following treatment with their respective stabilizers: Shield-1 for EL4/SCRAP cells and MLN7243 for EL4/NC NEDD8-GFP cells. GFP fluorescence after stabilizer treatment was much greater in EL4/SCRAP-GFP cells, as compared to EL4/NC NEDD8-GFP cells (Figure 6A). In contrast, total levels of GFP protein were virtually identical in both cell types, as determined by Western blot analysis (Figure 6B). To better quantify this observation, we determined the ratio of GFP fluorescence to total GFP protein in three independent experiments. The increase in GFP fluorescence was measured by subtracting the MFI of untreated cells from the MFI of the cell population in the presence of stabilizer, as determined by flow cytometry. The total amount of GFP protein in the cell lysate was quantified by densitometry and again the difference between untreated cells and cells treated with their respective stabilizer was recorded.

These values are tabulated and reported in Figure 6C. Using these values, we calculated the ratio of fluorescent GFP to total GFP protein for each cell type in each experiment. We found the ratio of fluorescent GFP to total GFP protein was nearly 5-fold greater in EL4/SCRAP-GFP cells compared to EL4/NC NEDD8-GFP cells. The change in fluorescence was not due to an artifact from treating cells with MLN7243 as treatment of EL4/SCRAP-GFP cells with MLN7243 resulted in an increase in GFP fluorescence in the absence of Shield-1 (Figure 6D). We also ensured that the intensity values of GFP measured by Western blot densitometry were within the linear range of detection by analyzing different molar concentrations of purified, recombinant GFP by Western blot. A representative blot is shown in Figure 6E and the values determined by densitometry plotted as a function of molarity (Figure 6F). The densitometry readings reported in Figure 6C fall within the range on GFP concentrations analyzed and these values are within the linear region of our standard curve (R^2^ = 0.9).

To determine if the loss of GFP fluorescence was simply due to the presence of NEDD8 appended to the N-terminus of GFP, we expressed both NC NEDD8-GFP and the cleavable NEDD8-GFP fusion proteins in E.coli and compared fluorescence to free GFP expressed in E. coli. GFP fluorescence was equivalent in E.coli strains transformed with either GFP or NEDD8-GFP fusion plasmids (Figure 6G) as was the total amount of protein as determined by Western blot analysis of GFP (Figure 6H). In E.coli, which lacks UCHL3 expression, NEDD8 remains fused to GFP even when the quadruple glycine residue is present. Therefore, the presence of NEDD8 on the N-terminus of GFP does not inherently prevent GFP-fluorochrome maturation. The 5-fold loss of fluorescence of NC NEDD8-GFP detected in EL4 cells indicates that NEDD8 fusion prevents GFP fluorescence in eukaryotic cells and suggests that the non-cleavable form of NEDD8 was preventing GFP from folding or otherwise preventing the fluorophore from maturing.

**Figure 7:**
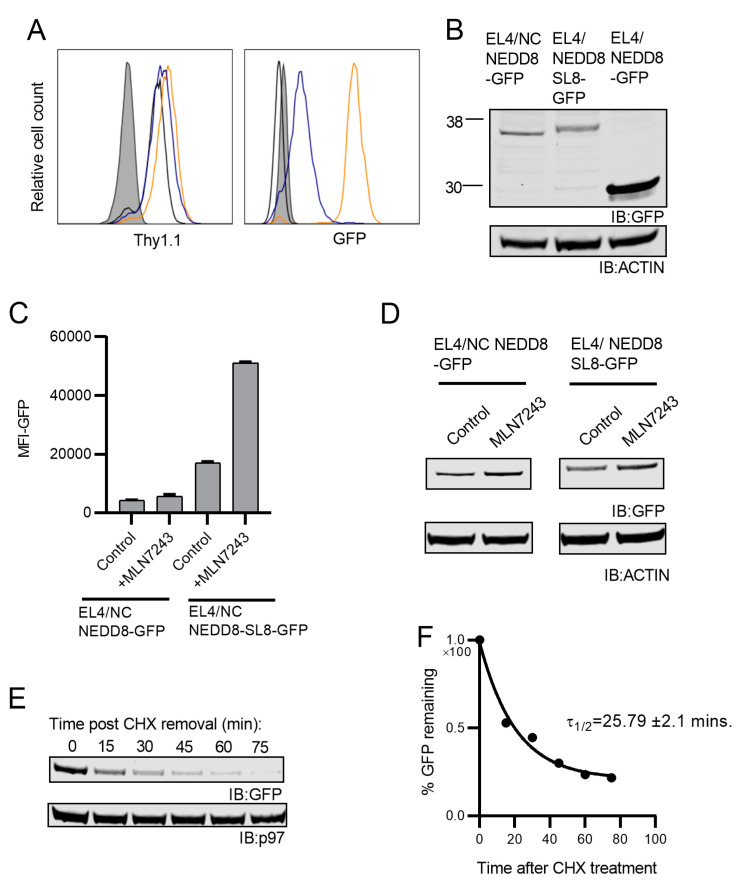
Fluorescence of NC NEDD8-GFP can be rescued by the addition of an eight-amino acid linker peptide. The construct NC NEDD8-SL8-GFP contains an eight-amino acid sequence (SIINFEKL) inserted between the terminal residue of NEDD8 and GFP. (**A**) Stable EL4 cell lines expressing NC NEDD8-GFP(black trace), NC NEDD8-SL8-GFP (blue trace), or NEDD8-GFP (orange trace) were analyzed by flow cytometry for Thy1.1 expression (left histogram) or GFP (right histogram). EL4 parental cells are shown in the shaded histogram. (**B**) EL4 cells expressing the three NEDD8-GFP constructs were analyzed for total GFP protein by Western blot with antibodies specific for GFP. (**C**,**D**) EL4/NC NEDD8-GFP or EL4/NC NEDD8-SL8-GFP cells were treated with MLN7243 to prevent protein degradation for 2 h and analyzed for GFP fluorescence by flow cytometry (**C**) or for total GFP protein by Western blot (**D**). (**E**) EL4/NC NEDD8-SL8-GFP cells were treated with CHX and cell lysates created at indicated times. Lysates were measured for total GFP by Western blot. Single phase exponential decay analysis was used to calculate the half-life of NC NEDD8-SL8-GFP from three independent experiments (**F**). All Western blots are cropped to show relevant bands.

The lack of GFP fluorescence in EL4 cells expressing NC NEDD8-GFP suggests that the fusion of NEDD8 prevents GFP from properly folding and the unfolded GFP is responsible for the rapid degradation of the construct. To test this, we generated an additional construct, termed NC NEDD8-SL8-GFP, which contains an eight-amino acid linker (SIINFEKL) between the terminal residue of NEDD8 and GFP. This peptide sequence is the same linker that separates the degradation signal in SCRAP from GFP. EL4 cells stably expressing this construct were generated and GFP fluorescence in each cell analyzed. NC NEDD8-SL8-GFP had more detectable fluorescence than the construct, which lacks the SL8 linker sequence (Figure 7A), though levels of GFP fluorescence are still far below the cleavable form of the construct. Western blot analysis demonstrated equivalent levels of total GFP in both EL4/NC NEDD8-GFP and EL4/NC NEDD8-SL8-GFP cells (Figure 7B). Both NC NEDD8-GFP and NC NEDD8-SL8-GFP are approximately the same size, indicating that NEDD8 remains fused to the construct containing the SL8 linker (Figure 7B). Furthermore, treating cells with MLN7243 to prevent protein degradation greatly increased the fluorescence of EL4/NC NEDD8-SL8-GFP cells while barely increasing the levels seen in EL4/NC NEDD8-GFP (Figure 7C). The levels of each protein were similar following MLN7243 treatment, as determined by Western blot (Figure 7D), indicating that the increase in GFP fluorescence is not due to an increase in total GFP fusion protein but rather due to the ability of GFP to properly fold when it is separated from N-terminal NEDD8 by a short peptide linker. Finally, CHX-chase experiments confirm that like NC NEDD8-GFP, NC NEDD8-SL8-GFP is rapidly degraded (Figure 7E,F). Taken together these data indicate that the N-terminal addition of NEDD8 results in the rapid degradation of the target protein irrespective of the ability of the target protein to fold properly.

### 3.6. Ubiquitination, but Not NEDDylation Is Required for SCRAP-GFP Degradation

**Figure 8:**
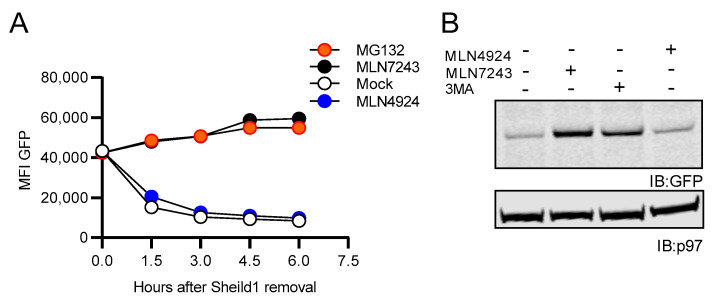
Ubiquitination, but not NEDDylation, is required for the rapid degradation of SCRAP-GFP. (**A**) EL4/SCRAP-GFP cells were treated with 2.5 μM Shield-1 overnight to allow accumulation of SCRAP-GFP. The cells were then washed, resuspended in fresh media, supplemented with either 10 μM MG132, 10 μM MLN7243, 10 μM MLN4924, or an equivalent volume of DMSO (mock), and incubated at 37 °C. GFP fluorescence was detected in each of the samples via flow cytometry every 1.5 h over 6 h and the MFI of the population reported. (**B**) Cell lysates of EL4 cells stably expressing NC NEDD8-GFP following a 3-h treatment with 10 μM MLN7243, 50 μM 3MA, and 10 μM MLN4924 were analyzed by Western blotting for GFP or p97. All Western blot images were cropped to show relevant bands.

To determine if NEDDylation was important for the destruction of an alternative rapidly degraded GFP construct, we treated EL4/SCRAP-GFP cells with Shield-1 overnight to create a pool of SCRAP-GFP. EL4/SCRAP-GFP cells were then washed to remove Shield-1 and cultured in media supplemented with either 10 μM MLN4924, 10 μM MLN7243, 10 μM MG132 or DMSO (mock). MLN4924 is a potent inhibitor of NAE1, the E1-activating enzyme for NEDD8 preventing NEDDylation of substrates [70]. GFP degradation in cells was then determined by flow cytometry every 1.5 h over 6 h (Figure 8A). Fluorescent SCRAP-GFP levels rapidly declined upon the removal of Shield-1 during mock treatment, in a proteasome-dependent fashion as previously published [57]. MLN7243 treatment resulted in a modest increase in GFP fluorescence over time, similar to proteasome-inhibition. This indicates an accumulation of SCRAP-GFP that likely requires ubiquitination prior to degradation. However, there was a rapid decrease in GFP fluorescence after treatment with MLN4924, indicating that neither the conjugation of NEDD8 to SCRAP-GFP nor the action of CRLs was necessary for degrading unfolded SCRAP-GFP. EL4/NC NEDD8-GFP cells were also treated with MLN4924 and compared to cells treated with either MLN7243 or 3-MA. Western blot analysis for GFP demonstrates that MLN4924 treatment did not result in an appreciable increase in GFP signal, whereas MLN7243 and 3-MA treatment did increase GFP protein levels as described above (Figure 8B). These data suggest that while fusion of NEDD8 to a protein results in protein degradation, endogenous cellular NEDDylation itself is not a necessity for degradation of GFP-containing constructs.

### 3.7. Non-Cleavable NEDD8-GFP Is Rapidly Degraded in an Ubiquitin-Dependent Manner in MCF7 Cells

**Figure 9:**
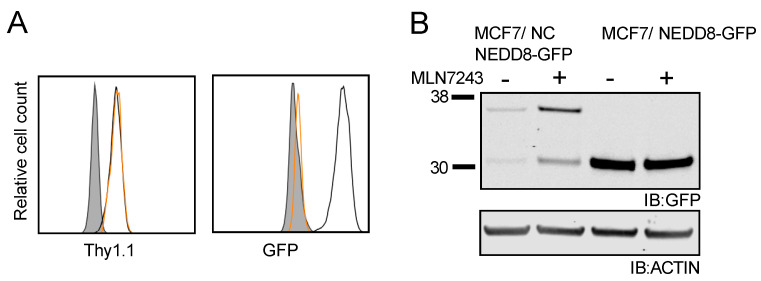
NC NEDD8-GFP requires ubiquitination for degradation in MCF7 cells. (**A**) MCF7 cells stably transfected with either NEDD8-GFP (black trace) or NC NEDD8-GFP (orange trace) vectors were analyzed by flow cytometry for Thy1.1 expression (left panel) and fluorescent GFP (right panel) compared to the parental cells (shaded histogram). (**B**) MCF7/NC NEDD8-GFP and EL4/NEDD8-GFP cells were treated with MLN7243 for 3 h and cell lysates analyzed by Western blot analysis. All Western blot images were cropped to show relevant bands.

Thus far, the work describing degradation of NEDD8-conjugated proteins has been conducted using EL4 cells. To confirm that this finding was not restricted to one cell type, we generated MCF7 cell lines stably expressing both NEDD8-GFP fusion proteins. Thy1.1 expression was virtually identical in both MCF7/NEDD8-GFP and MCF7/NC NEDD8-GFP cell lines, while GFP fluorescence was only detected in MCF7/NEDD8-GFP cells (Figure 9A). Similar to our results in the EL4 cell lines, a 28 kDa, GFP-reactive band was detected by Western blot in MCF7/NEDD8-GFP cells (Figure 9B), indicating that NEDD8 was processed and removed from the fusion protein. In contrast, a faint 36 kDa band was present when the non-cleavable form of NEDD8-GFP was expressed (Figure 9B). A barely detectable signal at the 28 kDa band in the MCF7/NC NEDD8-GFP lysate was present, similar to what was observed in EL4 cells. Treatment of MCF7 cells with MLN7243 resulted in an increase in signal of both the 36 and the 28 kDa band as was observed in EL4 cells. These data suggest that non-cleavable NEDD8-GFP is rapidly degraded in an ubiquitin-dependent manner in MCF7 cells, similar to EL4 cells, and that the recognition of NEDD8 bound to the N-terminus of a protein may act as a degron in different cell types.

## 4. Discussion

It has been nearly four decades since the discovery of ubiquitin and its role in protein degradation [71]. Despite its widely described role in protein degradation, post-translational modification with ubiquitin has many varied functions beyond protein degradation [72]. Contrastingly, post-translational modification with UBLs is thought to alter protein function or localization, but not to serve as a marker for protein degradation. Given that ubiquitin can act as both a signal for a protein’s degradation and a post-translational modification that alters a protein’s function, it is worth reconsidering non-canonical roles of UBLs. Here we find that appending the UBL NEDD8 to the N-terminus of a target protein leads to its degradation. Several studies have found that the N-terminus of a protein may be modified with ubiquitin, leading to the target proteins destruction [73,74,75,76], but no reports have mentioned the addition of NEDD8 to the N-terminus of a protein. However, NEDD8 can be acted upon by the ubiquitin E1 enzyme [77,78], and subsequently transferred like ubiquitin to target substrates of ubiquitin E3 ligases, a process termed atypical NEDDylation. Therefore, it is possible that NEDD8 may be appended to the N-terminus of a protein using the ubiquitin conjugation apparatus. This is most likely to occur when NEDD8 is overexpressed in diseases like glioblastoma and osteosarcoma [79,80]. While the goal of this project was to investigate if NEDD8, when fused to the N-termini of a model protein, was sufficient to induce degradation, these findings may be relevant in disease where NEDD8 expression is dysregulated.

Destruction of NEDD8-conjugated proteins could occur through multiple possible mechanisms. NUB1 is a protein with a NEDD8-interacting domain that associates with the proteasome and has been previously identified as a critical component for the destruction of NEDD8-modified proteins [50]. We find that levels of non-cleavable NEDD8-GFP rise following siRNA depletion of NUB1, similar to treatment with proteasome inhibitors. However, the amount of non-cleavable NEDD8-GFP rescued from degradation is not as robust as when cells are treated with MLN7423, an inhibitor of UBE1. MLN7243 treatment eliminates a cell’s ability to conjugate ubiquitin to various substrates and prevents protein degradation. MLN7243 treatment greatly enhanced levels of non-cleavable NEDD8-GFP, more so than proteasome inhibition or the inhibition of the autophagy pathway, demonstrating that degradation of non-cleavable NEDD8-GFP requires a functional ubiquitin system. However, we did not isolate poly-ubiquitinated forms of NC NEDD8-GFP from EL4 cell lysates. A slight signal, which may be NC-NEDD8-GFP containing 2 ubiquitin molecules, was detected in the analysis but this must be treated with caution as there is an apparent affinity for NC NEDD8-GFP for the TUBES and this higher molecular weight band may be some other type of post-translational protein modification. These results suggest that NC NEDD8-GFP is not poly-ubiquitinated prior to its destruction and the ubiquitin conjugation system may be necessary for other cellular functions. One possible explanation is that the model protein is degraded following mono-ubiquitination of the substrate (or the addition of multiple ubiquitin monomers to a substrate), as has been reported for other substrates [16,18,81,82,83,84]. Ubiquitination may also regulate the activity of yet another enzyme involved in the degradation of NC NEDD8-GFP. Interestingly, ubiquitination of NUB1 by Mdm2 is necessary for proper NUB1 function [85]. Therefore the MLN7243 treatment may inhibit protein degradation through multiple pathways.

Despite the rapid degradation of the non-cleavable fusion of NEDD8-GFP, a very faint band corresponding to the size of native GFP was detected in Western blot lysates (Figure 1D), which did not co-stain for NEDD8. This band became more pronounced upon either proteasome or ubiquitination inhibition, but not upon inhibition of autophagy. Interestingly, a slight increase in GFP fluorescence was also seen upon proteasome and ubiquitination inhibition but not autophagy. While the bulk of NC NEDD8-GFP appears to be rapidly degraded, these data suggest that NEDD8 is removed from a small fraction of GFP resulting in the 28 kDa band. It is likely this is the fraction of protein accounts for the low level of GFP fluorescence. These data suggest that when NEDD8 is physically bound to the N-terminus of the protein, GFP is unable to fold and gain fluorescence. This hypothesis is in alignment with the data of Figure 6 showing that despite an increase in GFP signal by Western blot of the NC NEDD8-GFP molecule, only a modest increase in fluorescence is detected when ubiquitination is inhibited. If true, then the rapid degradation of NEDD8-modified constructs may stem from the misfolded nature of the target protein and have nothing to do with NEDD8 acting as a signal for degradation. To control for this concern, we inserted eight additional amino acids between NEDD8 and GFP and found the resulting fusion protein to be both fluorescent and rapidly degraded (Figure 7). Therefore, while the presence of NEDD8 at the N-terminal of a protein can certainly impact the proteins’ ability to fold, NEDD8 can serve as a signal for degradation irrespective of the folded or unfolded state of the modified protein. Interestingly, the potential misfolding of NC-NEDD8-GFP may highlight an alternative role for NUB1, namely its importance in promoting the degradation of misfolded proteins [48]. If NC-NEDD8-GFP does in fact fail to properly fold in the cell, then NUB1 may be necessary for its degradation, which is consistent with our data showing NUB1 depletion elevated levels of NC-NEDD8-GFP. Alternatively, the misfolding of the substrate we detect in our model protein may create a situation that favors the removal of the misfolded protein by the autophagy pathway. While a few recent reports have demonstrated proteasome-mediated degradation of NEDDylated substrates [47,48,49], there is less evidence in the literature that NEDDylated proteins can be degraded by autophagy [86]. Future work will have to carefully evaluate if autophagy-mediated degradation of naturally NEDDylated proteins occurs or if this is an artifact of the misfolded nature of the model substrate.

Previous reports have demonstrated that fusion of NEDD8-to the C-terminus of GFP does not result in loss of GFP fluorescence [48,87], which demonstrates that the presence of NEDD8 itself does not impact GFP folding, maturation, and fluorescence but rather that the N-terminal NEDD8 modification impacts fluorescence. Other published reports have indicated that upon proteasome inhibition, non-cleavable, N-terminally fused ubiquitin does not impair GFP fluorescence [88], which suggests that the presence of a non-cleavable UBL directly adjacent to GFP does not impact GFP folding and maturation. Furthermore, expression of NC NEDD8-GFP in prokaryotic cells results in equivalent fluorescence to GFP alone, which suggests that the fusion of NEDD8 to the N-terminus of GFP alone does not intrinsically impact the ability of the fluorochrome to mature, but rather in eukaryotic cells, N-terminal addition of NEDD8 somehow prevents the fluorochrome from maturing. How eukaryotic cells recognize N-terminal NEDDylation and prevent the substrate from properly folding is unknown but should be the subject of future research.

Our findings that N-terminal fusion of NEDD8 initiates the destruction of a protein are similar to other findings that both ubiquitin and UBL N-terminal fusion can result in protein degradation. DNA constructs encoding N-terminal ubiquitin moieties fused in frame with a target protein were proposed to be the ubiquitin monomer that the poly-ubiquitin chain would “grow” from [74,75,76,88]. However, recent reports demonstrate that removal of lysine residues within the ubiquitin region of the fusion protein does not prevent protein degradation, suggesting that an N-terminal addition of ubiquitin alone can result in the accumulation of ubiquitin chains on the remainder of the fusion protein [52]. Fusion of another UBL, FAT10, to GFP results in its accelerated destruction by the proteasome and this too is believed to occur independently of the addition of ubiquitin to the lysine residues of FAT10 [24]. It is therefore difficult to know if the ubiquitin/UBL moiety itself serves as the scaffold for the addition of poly-ubiquitin chains, or if the presence of ubiquitin/UBL alters the targeted protein in some other manner, which renders it the substrate of ubiquitination. While it is tempting to speculate that the simple addition of any UBL to the N-terminus of a protein can act as a degron, the fusion of the UBL SUMO-1 did not induce GFP degradation [24] and ISG15 fusion to a viral nucleoprotein did not enhance proteasome-mediated degradation [89] when compared to either ubiquitin or FAT10 fusion.

## 5. Conclusions

In summary, we find that the covalent addition of NEDD8 to the N-terminus of a protein acts as a degradation signal that requires an active ubiquitination system. Though the addition of NEDD8 does lead to the destruction of a target protein in our model system, this may not be a universally utilized mechanism in the cell, as the degradation of another destabilized protein occurred in a ubiquitin-dependent manner, but was largely unaffected by the loss of NEDD8 activity within the cells. As new substrates of NEDD8 are identified within cells, it is worth considering if the addition of NEDD8 acts to modify the function of a protein or as a signal for the substrate’s destruction.

## Data Availability

The datasets used and/or analyzed during the current study are available from the corresponding author on reasonable request.
